# Supplementary material for: Robust ultrathin nanoporous MOF membrane with intra-crystalline defects for fast water transport
Source: Nat Commun. 2022 Jan 11;13:266. doi: 10.1038/s41467-021-27873-6 (PMC8752604; doi:10.1038/s41467-021-27873-6)
Supplement: Supplementary file 1 — Supplementary Information [file 41467_2021_27873_MOESM1_ESM.pdf]

Supplementary Information

Robust ultrathin nanoporous MOF membrane with intra-crystalline defects for fast water transport

Xueling Wang [1], Qiang Lyu [2], Tiezheng Tong [3], Kuo Sun [1], Li-Chiang Lin [2], Chuyang Y. Tang [4], Fenglin Yang [1], Michael D. Guiver [*5], Xie Quan [*1], Yingchao Dong [*1]

[1] Key Laboratory of Industrial Ecology and Environmental Engineering (Ministry of Education, MOE), School of Environmental Science and Technology, Dalian University of Technology, Dalian, Liaoning 116024, China

[2] William G. Lowrie Department of Chemical and Biomolecular Engineering, The Ohio State University, Columbus, Ohio 43210, United States

[3] Department of Civil and Environmental Engineering, Colorado State University, Fort Collins, Colorado 80523, United States

[4] Department of Civil Engineering, The University of Hong Kong, Pokfulam, Hong Kong, China

[5] State Key Laboratory of Engines, and Collaborative Innovation Center of Chemical Science and Engineering (Tianjin), Tianjin University, Tianjin 300072, China

Correspondence to:

Prof. Dr. Michael D. Guiver, E-mail: michael.guiver@outlook.com

Prof. Dr. Xie Quan, E-mail: quanxie@dlut.edu.cn

Prof. Dr. Yingchao Dong, E-mail: ycdong@dlut.edu.cn

Table of Contents

Supplementary Note 1: Experimental and Characterization 3

S1.1 Raw Materials and Chemical Reagents 3
Supplementary Table 1 3
S1.2 Fabrication of Substrate 4
Supplementary Fig. 1 4
S1.3 Schematic of the PV simulation system 5
Supplementary Fig. 2 6
S1.4 Characterization 7
S1.5 Pore Size Distribution 8
Supplementary Table 2 8
S1.6 Isosteric heat of water adsorption 9
S1.7 Measurements of water permeability, adsorption coefficient and diffusivity coefficient[20] 10

Supplementary Note 2: Results and Discussion 12

S2.1 Characterization of Sol and γ-Al_2O_3 Interlayer 12
Supplementary Fig. 3 12
S2.2 Optimization of the γ-Al_2O_3 Interlayer 13
Supplementary Fig. 4 14
Supplementary Fig. 5 15
Supplementary Fig. 6 16
Supplementary Fig. 7 17
Supplementary Fig. 8 18
Supplementary Fig. 9 19
S2.3 Optimization and Performance of ML-UiO-66 Membranes 20
Supplementary Fig. 10 20
Supplementary Fig. 11 22
Supplementary Fig. 12 23
Supplementary Fig. 13 24
Supplementary Table 3 25
Supplementary Fig. 14 26
Supplementary Fig. 15 27
Supplementary Table 4 28
Supplementary Table 5 29
Supplementary Fig. 16 30
Supplementary Fig. 17 31
Supplementary Table 6 32
Supplementary Fig. 18 33
Supplementary Fig. 19 33
Supplementary Fig. 20 34
Supplementary Fig. 21 35
Supplementary Fig. 22 36
Supplementary Table 7 37
Supplementary Table 8 38
Supplementary Fig. 23 39
Supplementary Table 9 40
S2.4 Characterization of Missing-linker Defect 42
Supplementary Fig. 24 42
Supplementary Table 10 43
Supplementary Fig. 25 45
Supplementary Fig. 26 46
S2.5 Computational Modeling 47
Supplementary Fig. 27 47
Supplementary Fig. 28 48
Supplementary Fig. 29 49
Supplementary Fig. 30 50

Supplementary References 51

Supplementary Note 1: Experimental and Characterization

S1.1 Raw Materials and Chemical Reagents

Supplementary Table 1 Raw materials and chemical reagents.

Material	Molecular formula	Purity	Manufacturer
Zirconium chloride	$ZrCl_4$	99.9%	Aladdin, China
Aluminium isopropoxide	$C_9H_{21}AlO_3$	AR	Aladdin, China
1,4-Dicarboxybenzene	H_2BDC	99%	Aladdin, China
Polyvinyl alcohol (PVA) (Molecular weight (MW) = 1750±50)	$(C_2H_4O)_n$	AR	Sinopharm Chemical Reagent Co., Ltd., China
Polyvinylpyrrolidone (PVP) (MW = ~30,000)	$(C_6H_9NO)_n$	AR	Sinopharm Chemical Reagent Co., Ltd., China
Sodium chloride	NaCl	AR	Damao Chemical Reagent Factory, Tianjin, China
N-methylpyrrolidone (NMP)	C_5H_9NO	AR	Damao Chemical Reagent Factory, Tianjin, China
Acetic acid	CH_3COOH	AR	Damao Chemical Reagent Factory, Tianjin, China
N,N-dimethylformamide (DMF)	C_3H_7NO	AR	Damao Chemical Reagent Factory, Tianjin, China
Nitric acid	HNO_3	AR	Damao Chemical Reagent Factory, Tianjin, China
Ethanol	C_2H_5OH	AR	Damao Chemical Reagent Factory, Tianjin, China
3 % mol Yttrium oxide stabilized tetragonal zirconia polycrystal	ZrO_2, $Zr_{0.9}Y_{0.1}O_{1.95}$	High-purity	Fanmeiya Co., Ltd., China
Polyethersulfone (PES) (MW = ~100,000)	$(C_{12}H_8O_3S)_n$	AR	BASF, Germany

S1.2 Fabrication of Substrate

All the reagents (Supplementary Table 1) in our study were used without any further purification. An ultra-pure water system (GWA-UN, Purkinje General, China) was used for preparing saline solutions. Robust hollow fiber ZrO_2 (3Y-TZP, 3 % mol yttrium oxide stabilized tetragonal zirconia polycrystal) ceramic substrates were first fabricated by an immersion-induced phase inversion process (i.e., dry-wetting spinning method) followed by sintering at 1100°C for 4 h in a high temperature furnace (GR. BF40/18, Shanghai Guier Machinery Equipment Co., Ltd., China) (Supplementary Fig. 1)[1-4]. For such ZrO_2 substrates, a sandwich hierarchical pore structure was quantitatively produced, allowing a reduction in transport resistance while maintaining sufficient mechanical stability (Fig. 1).

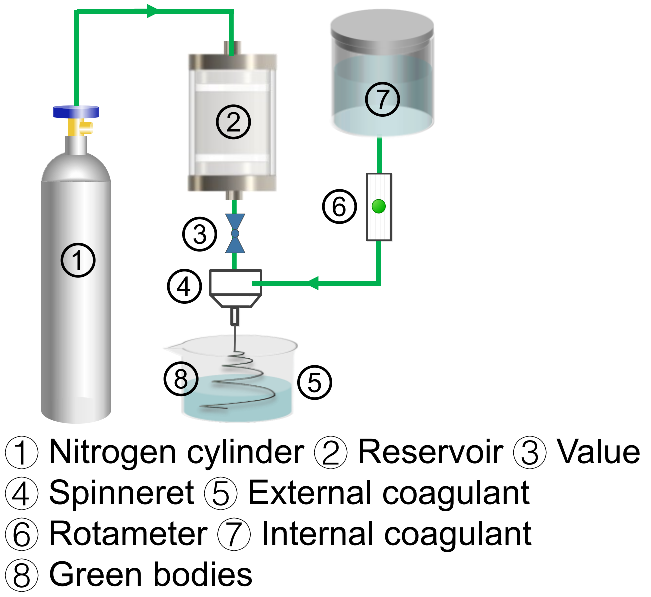

Supplementary Fig. 1 Schematic diagram of fabrication of ZrO_2 substrates via a dry-wetting spinning method.

S1.3 Schematic of the PV simulation system

The PV simulation system, as depicted in Supplementary Fig. 2, consists of saline solution on the feed side and a vacuum space on the permeate side, separated by a UiO-66/ML-UiO-66 membrane (i.e., as an active PV membrane layer) with a thickness of ~25 Å. Such sandwich simulation setup was also adopted in prior studies by some of us to study PV ethanol/water separation[5,6]. The membrane surface was parallel to the [1 1 1] plane of its crystal structure[7]. The open metal sites resulting from the surface cleavage and missing-linker defects were saturated with acetate group (CH_3COO-)[8,9]. The saline solution containing 20 Na^+/Cl^- ion pairs solvated by 2000 water molecules, which was bounded by a graphene sheet as a piston to modulate the pressure at 1 atm. The vacuum condition on the permeate side was also maintained using a graphene sheet as an adsorbing plate to capture all permeated molecules. Each studied active membrane layer (containing hundreds of atoms) was relaxed by the conjugate gradient method using the Forcite module in the Materials Studio package. The convergence criteria for energy, force, and displacement were set to be 2×10^{-5} kcal mol^{-1}, 0.001 kcal mol^{-1} Å^{-1}, and 1×10^{-5} Å, respectively. For simplicity, these structures have the same cell parameter as reported in an experimental work[10]. Periodic boundary conditions were applied, and an additional 50 Å-thick vacuum region along z-direction was included to reduce the self-interactions between periodic images.

In these calculations, 12−6 Lennard-Jones (L-J) potential and long-range Coulombic interactions with static point charges were used to describe non-bonded interactions. L-J parameters of the framework atoms were taken from the DREIDING force field[11] except for zirconium atoms whose parameters were instead from the universal force field (UFF)[12]. Atomic charges of the framework atoms were derived from the PM7 calculations based on population analysis[13,14]. The rigid SPC/E model[15] with the SHAKE algorithm was used to model water molecules. Non-bonded potentials developed by Joung et al.[16] were used for salt ions. The Lorentz-Berthelot mixing rule

was applied to estimate the cross pairwise L-J parameters between atoms. However, since the UiO-66 membranes should not be influenced by the piston and the adsorbing plate, their pairwise L-J coefficients (i.e., piston-membrane and plate-membrane) were set to zero. The L-J potentials were truncated and shifted to zero at a cutoff distance of 12 Å, while the long-range Coulombic interactions were computed using the particle-particle-particle-mesh (PPPM) algorithm with a precision of 10^{-6}.

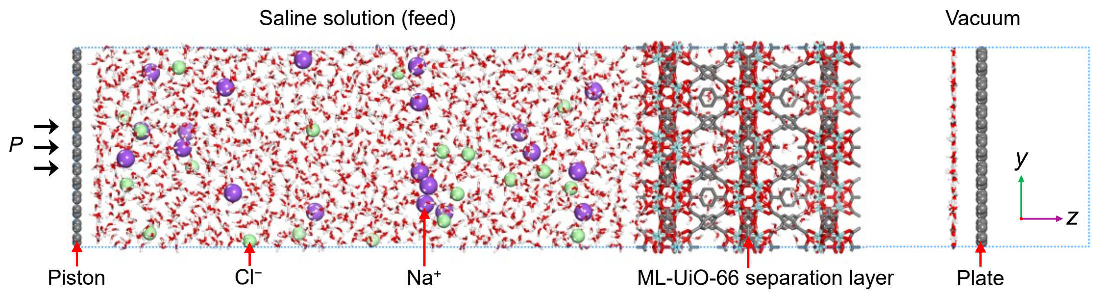

Supplementary Fig. 2 Schematic illustration of the PV simulation system. Membrane structure and water molecules are represented as sticks with color codes of carbon: grey, oxygen: red, hydrogen: white, and zirconium: cyan. Salt ions, piston, and plate are represented as CPK balls with color codes of carbon: grey, oxygen: red, hydrogen: white, sodium: purple, and chloride: green. Hydrogen atoms of the framework structure are omitted for clarity.

S1.4 Characterization

The crystalline phases of ML-UiO-66 powders and ML-UiO-66 membranes were identified by an X-ray diffraction (XRD) instrument (SmartLab 9KW, Rigaku Corporation, Japan) using Cu Ka (λ = 1.5418 Å) radiation (voltage = 240 kV, current = 50 mA) in the 2θ range of 2 − 80° at a scanning rate of 20° min^{-1}. Structural morphologies of UiO-66/ML-UiO-66 membranes were characterized by field emission scanning electronic microscopy (FE-SEM) (NOVA NanoSEM 450, FEI company, USA) equipped with energy dispersive spectrometer (EDS) (X-Max50, Oxford, UK) at an acceleration voltage of 3 kV after platinum coating for 30 s. The nitrogen adsorption-desorption isotherms of the UiO-66 and ML-UiO-66 powders were recorded by an automatic physical adsorption apparatus (AS-1-MP-11, Quantachrome, USA) at -196°C (liquid nitrogen bath) after vacuum activation (~60 Pa) at 180°C for 4 h. Then the pore size distribution was calculated by Horvath-Kawazoe method. Thermal analysis of both UiO-66 and ML-UiO-66 powders was determined by a thermogravimetric/different thermal analyzer (TG/DTA 6300, Seiko Co., Ltd, Japan). ATR-FTIR spectra were recorded by Attenuated total reflectance-Fourier transform infrared spectroscopy (EQUINOX55, Bruker, Germany) in the wavelength range of 4000 − 500 cm^{-1}. ^1H NMR spectrum was recorded using a nuclear magnetic resonance spectrometer (Bruker Advance III500, Bruker, Switzerland)[17]. The pore size distributions of ZrO_2 and $ZrO_2@\gamma\text{-}Al_2O_3$ substrates were characterized by a membrane porometer (PSMA-10, GaoQian Functional Materials Co., Ltd, China). The surface hydrophobic/hydrophilic properties of ZrO_2 and $ZrO_2@\gamma\text{-}Al_2O_3$ substrates, UiO-66 and ML-UiO-66 membranes were measured in air using water droplets by a contact angle instrument (SL200KB, Shanghai Suolun, China). The surface roughness of ZrO_2 and $ZrO_2@\gamma\text{-}Al_2O_3$ substrates, UiO-66 and ML-UiO-66 membranes were characterized by atomic force microscope (AFM) (Dimension ICON, Bruker, USA). Surface chemical characterization of ZrO_2 and $ZrO_2@\gamma\text{-}Al_2O_3$ substrates was performed by X-ray photoelectron spectroscopy (XPS) (K-Alpha+, Thermo Fisher, UK). Water vapor

adsorption ability of UiO-66 and ML-UiO-66 structures was obtained using an intelligent gravimetric analyzer (dynamic vapor sorption (DVS) standard, Surface Measurement System Ltd., UK). The mechanical strength of the ZrO_2 substrate was measured using the three-point bending method by a universal testing machine (AGS-10KN, Shimadzu Ltd., Japan). The bulk porosity of ZrO_2 substrate and γ-Al_2O_3 interlayer was measured by the gravimetric method while surface porosity was calculated by Image J software based on their surface FE-SEM images. The porosity of UiO-66 and ML-UiO-66 membranes was simulated by zeo++ software.

S1.5 Pore Size Distribution

The mean pore size and pore size distribution of UiO-66 and ML-UiO-66 membranes were experimentally determined by the solute rejection method using a laboratory-made dead-end filtration setup[17,18]. A series of ~200 ppm PEG or glycerol solutions (molecular weights: 92, 200, 400 and 600 g mol^{-1}) were used as the feed solution. The operation transmembrane pressure was 3 bar. The concentration of feed (C_f, ppm) and permeate (C_p, ppm) were measured using a total organic carbon analyzer (TOC/TN analyzer, multi N/C 2100S, Germany). The effective solute rejection (R', %) was calculated by Supplementary Eq. 1:

$$R' = \left(1 - \frac{C_p}{C_f}\right) \times 100\% \quad (1)$$

Supplementary Table 2 Molecular weight and Stokes diameters of the organic solutes.

**Solute**	**Molecular weight M_w (g mol^{-1})**	**d_s (nm)**
Glycerol	92	0.52
PEG 200	200	0.64
PEG 400	400	0.94
PEG 600	600	1.18

Based on molecular weights, the dependence of Stokes diameters (d_s, nm) on molecular weight M_w (g mol^{-1}) is shown as Supplementary Eq. 2:

$$d_s = 33.46 \times 10^{-12} \times M_w^{0.557} \tag{2}$$

Then, the solute rejection was plotted against Stokes diameter on a log-normal probability diagram and linear regression was performed. The mean effective pore diameter (μ_p, nm) is the size of a solute where its rejection is 50%. The geometric standard deviation (σ_p) of a membrane is the size ratio of solutes with rejections of 84.13% and 50%. The pore size distribution of the membrane was generated using Supplementary Eq. 3:

$$\frac{dR(d_p)}{d(d_p)} = \frac{1}{d_p \ln\sigma_p \sqrt{2\pi}} \exp\left\{-\frac{\left(\ln d_p - \ln\mu_p\right)^2}{2\left(\ln\sigma_p\right)^2}\right\} \tag{3}$$

where d_p (nm) is the effective pore diameter.

S1.6 Isosteric heat of water adsorption

The isosteric heat of adsorption can be determined by the Clausius-Clapeyron equation as Supplementary Eq. 4:

$$Q_{st} = -\mathrm{R}T^2 \left(\frac{\partial \ln Pp}{\partial T}\right)_\theta \tag{4}$$

where Q_{st} (kJ mol^{-1}) is the isosteric heat of adsorption, R (8.314 J mol^{-1} K^{-1}) is the gas content, T (K) is the absolute temperature, P (bar) is the pressure, and θ (%) is the sorbed amount. Integration of the Clausius-Clapeyron equation (Supplementary Eq.4) gives Supplementary Eq. 5:

$$\ln p = \frac{Q_{st}}{\mathrm{R}T} + C \tag{5}$$

In this study, adsorption isotherm data measured at 298.15 K and 303.15 K was used to obtain the plots of adsorption heat. The adsorption heat at a given uptake was calculated from the fitted slopes of the Supplementary Eq. 5[19].

S1.7 Measurements of water permeability, adsorption coefficient and diffusivity coefficient[20]

For the water adsorption coefficient measurements, the UiO-66 and ML-UiO-66 membranes were immersed into DI water for 2 days to fully hydrate the membranes. Then, any water droplets on the membrane surface were gently removed with a dry tissue and the wet membrane (W_{wet}, g) was quickly weighed using an analytical balance. After drying in a vacuum oven at 80°C for 24 h, the dry membrane (W_{dry}, g) was weighed.

The volume fraction of water is related to the water adsorption coefficient, K_w(-). The K_w(-) is defined as Supplementary Eq. 6:

$$K_w = \frac{(W_{wet} - W_{dry})/\rho_w}{(W_{wet} - W_{dry})/\rho_w + V_{dry}} \quad (6)$$

where V_{dry} (cm^3) is the volume of the dry membrane, ρ_w (g cm^{-3}) is the density of water.

The hydraulic water permeability (P_w^H, L μm m^{-2} h^{-1} bar^{-1}) was measured using DI water as a feed at room temperature (25°C) during PV process. The hydraulic water permeability was calculated by Supplementary Eq. 7:

$$P_w^H = \frac{\Delta V}{A\,\Delta t}\frac{l}{\Delta P} \quad (7)$$

where ΔV (L) is the volume of the permeated water, A (m^2) is the effective membrane areas, Δt (h) is the test time interval, l (μm) is the hydrated membrane thickness, and ΔP (bar) is the transmembrane pressure difference.

Like the solution–diffusion mechanism in polymeric membranes, water molecules transported through UiO-66 and ML-UiO-66 membranes following the adsorption–diffusion mechanism during PV process. The intrinsic hydraulic water permeability (P_w, cm^2 s^{-1}) of a membrane is associated with P_w^H, which is calculated as Supplementary Eq. 8:

$$P_{\mathrm{w}} = D_{\mathrm{w}}\, K_{\mathrm{w}} = P_{\mathrm{w}}^{\mathrm{H}} \frac{\mathrm{R}T}{V_{\mathrm{w}}} \tag{8}$$

where D_{w} (-) is the water adsorption coefficient, R (8.314 J mol^{-1} K^{-1}) is the ideal gas constant, and T (K) is the absolute temperature during the permeability measurements.

Supplementary Note 2: Results and Discussion

S2.1 Characterization of Sol and γ-Al_2O_3 Interlayer

Supplementary Fig. 3a shows that the D_{50} particle size of the prepared boehmite sol is ~70 nm with the majority between 15 and 105 nm. The thermogravimetric (TG) results of boehmite sol show that there is a total mass loss of ~49 wt.% with two stages and two exothermic peaks (Supplementary Fig. 3b). A mass loss of 9.7 wt.% between 25 and 140°C can be ascribed to the loss of adsorbed water while a larger mass loss of 39.3 wt.% between 140 and 600°C can be ascribed to the decomposition of aluminum hydroxide and organics[21]. The XRD pattern indicates the formation of γ-Al_2O_3 phase (JCPDS#29-0063) when sintered at 750°C for 2 h (Supplementary Fig. 3c)[21,22]. The diffraction peaks at 37.6°, 45.8° and 66.7° can be assigned to the (311), (400) and (440) crystalline planes of γ-Al_2O_3 respectively. Supplementary Fig. 3d shows that a defect-free γ-Al_2O_3 interlayer with much finer pores was formed on the ZrO_2 substrate.

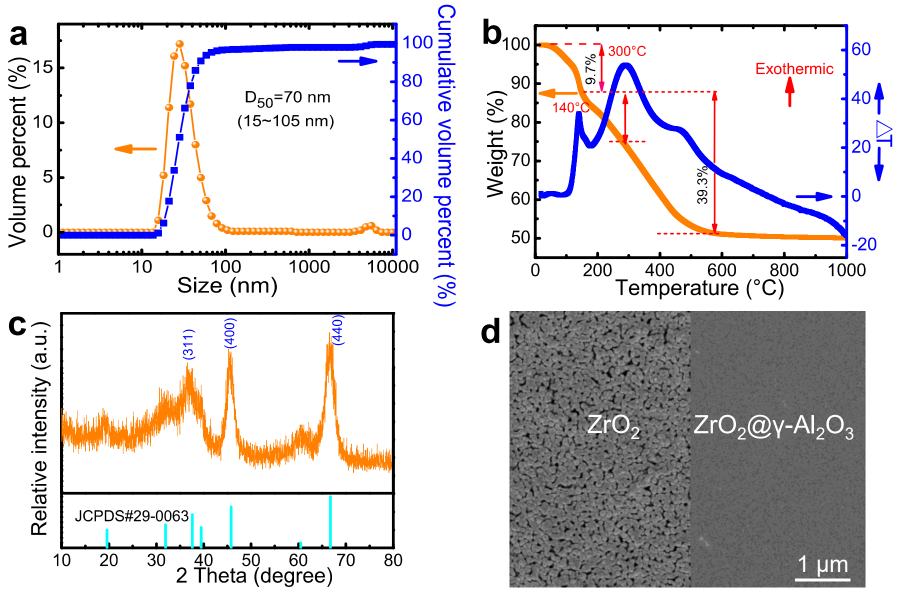

Supplementary Fig. 3 Particle size distribution **(a)** and TG-DTA curves **(b)** of boehmite sol. **(c)** XRD pattern of nanoporous γ-Al_2O_3 interlayer after sintering at 750°C for 2 h. **(d)** Surface FE-SEM morphology of ZrO_2 and ZrO_2@γ-Al_2O_3 substrates.

S2.2 Optimization of the γ-Al_2O_3 Interlayer

Supplementary Fig. 4 shows that defect-free nanoporous γ-Al_2O_3 interlayer could be obtained and optimized via a simple dip-coating and sintering technique. The relationship between the thickness of the γ-Al_2O_3 interlayer and coating time fits well with a classical dip-coating model (Supplementary Fig. 4h, Supplementary Eq. 9)[23].

$$L_{\mathrm{m}}=2\left(\frac{\varepsilon_S\,\gamma\,K_{\mathrm{m}}}{\eta\,\alpha\,\mathrm{R}}\,t\right)^{1/2} \tag{9}$$

where L_m (m) is the thickness of interlayer membrane, ε_s is the porosity of interlayer membrane, γ (N m^{-1}) is the interfacial tension of interlayer membrane, K_m (m^2) is the permeability of interlayer membrane, η (N m^{-2}) is the viscosity of the dispersion liquid, $\alpha = (\psi_0/\psi_m)$-1 (ψ_0 and ψ_m are the volume fraction of the particles in the suspension and in the wet membrane respectively), t (s) is the dip-coating time and R (m) is the radius of the assumed cylindrical pores.

The pure water flux of ZrO_2@γ-Al_2O_3 membrane is positively proportional to transmembrane pressure for different coating times (1 – 5 s) (Supplementary Fig. 4i), which is consistent with the classical viscous flow model[23].

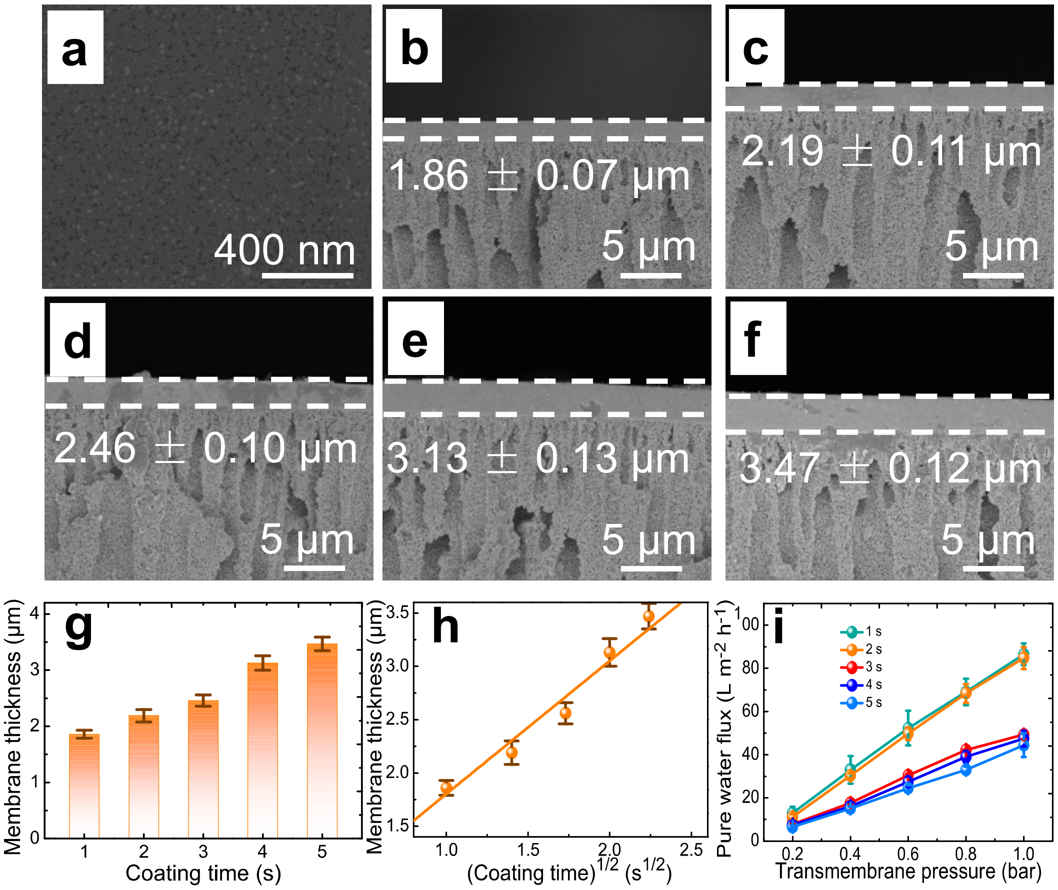

Supplementary Fig. 4 Optimization of the γ-Al_2O_3 interlayer. (a-f) Surface FE-SEM image, cross-sectional FE-SEM images at different coating times (a, b: 1 s, c: 2 s, d: 3 s, e: 4 s, f: 5 s). **(g)** The relationship between interlayer membrane thickness and coating time. **(h)** Fitted linear relationship between interlayer membrane thickness and square root of coating time and **(i)** pure water flux vs. transmembrane pressure for the ZrO_2@γ-Al_2O_3 membranes fabricated with different coating times. (Numbers that follows the ±signs are standard deviation (SD) in this study.)

Supplementary Fig. 5 shows the pore size distribution and average pore size of ZrO_2 (142 ± 48 nm) and ZrO_2@γ-Al_2O_3 (48 ± 1 nm) substrates.

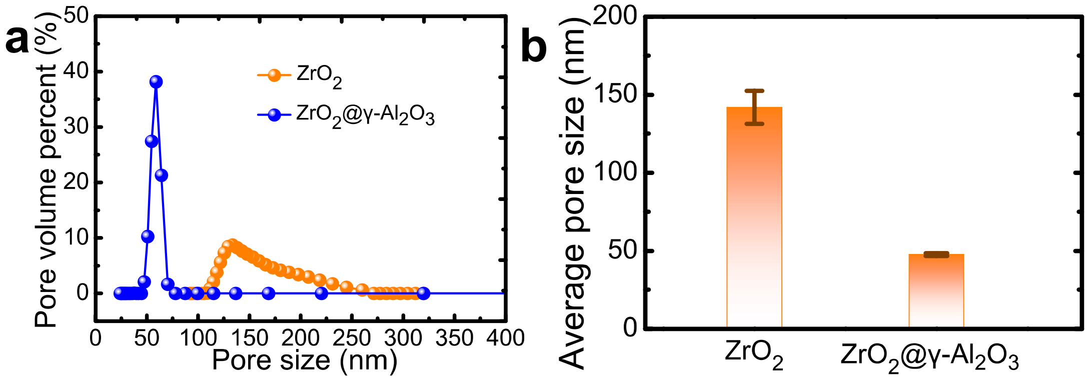

Supplementary Fig. 5 (a) Pore size distribution and (b) mean pore size of ZrO_2 and ZrO_2@γ-Al_2O_3 substrates. (Numbers that follows the ±signs are standard deviation (SD) in this study.)

Supplementary Fig. 6 shows that after introducing the γ-Al_2O_3 nanoporous interlayer, the ZrO_2@γ-Al_2O_3 substrate has two-fold higher Al-OH group concentration than ZrO_2 substrate, which provides more heterogeneous nucleation site for the growth of ML-UiO-66 membranes.

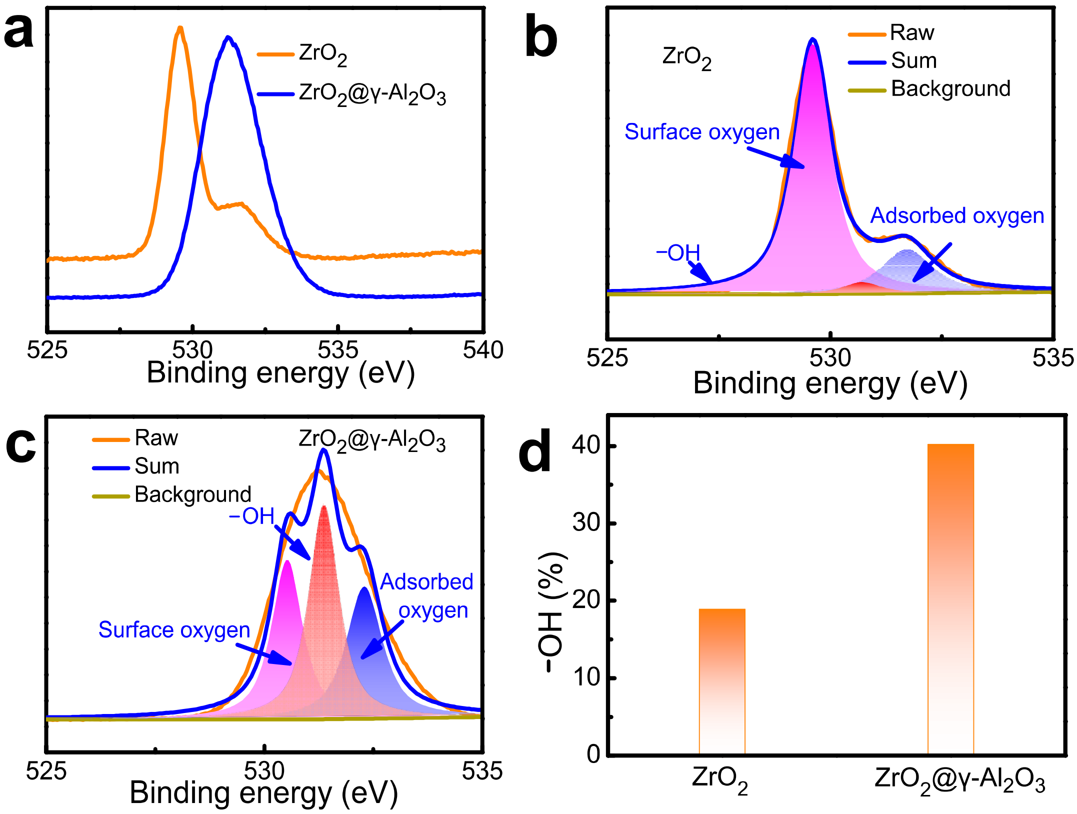

Supplementary Fig. 6 (a) XPS O1s survey of ZrO_2 and ZrO_2@γ-Al_2O_3 substrates, high resolution XPS O1s survey of ZrO_2 substrate **(b)** and ZrO_2@γ-Al_2O_3 substrate **(c)**, **(d)** Normalized intensity in surface –OH groups of ZrO_2 and ZrO_2@γ-Al_2O_3 substrates.

The surface properties of substrate are the dominant factor for crystalline MOF membrane growth as the growth of MOF crystals first occurs on the solid-liquid interface (i.e., substrate–solution interface). Specially, besides surface –OH groups, surface roughness also plays an important role in heterogeneous nucleation and subsequent kinetic growth of ML-UiO-66 crystals. This is by providing different energy barriers, which affect the final ML-UiO-66 membrane features, such as integrity (inter-crystalline defects), thickness, and surface roughness. Supplementary Fig. 7 shows that the $ZrO_2@\gamma$-Al_2O_3 substrate has a much lower surface roughness (R_a = ~10 ± 2 nm) than ZrO_2 substrate (R_a = ~27 ± 3 nm).

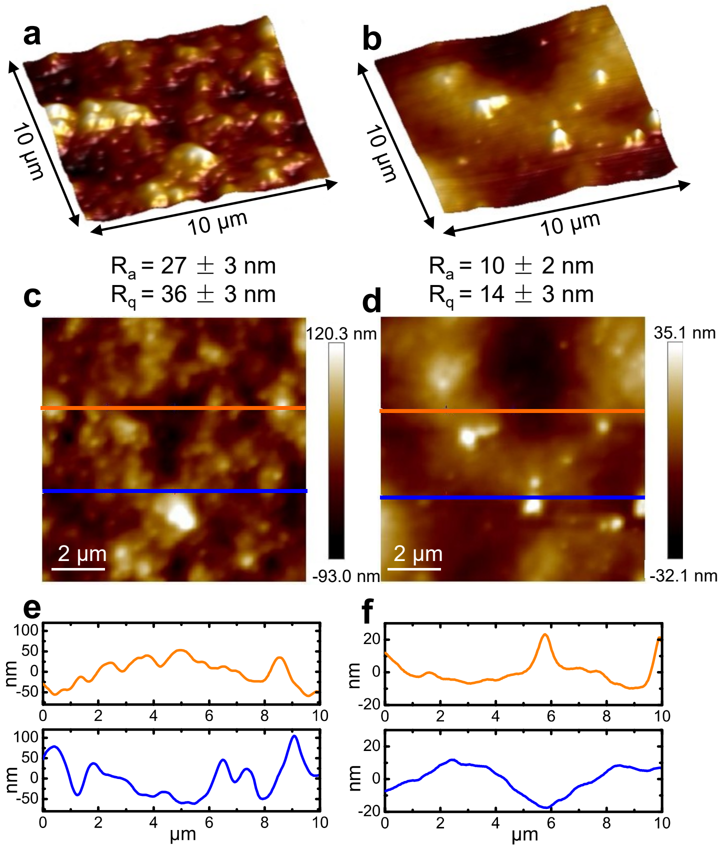

Supplementary Fig. 7 3D AFM images (with R_a (average roughness) and R_q (root-mean-square roughness)) of ZrO_2 **(a)** and $ZrO_2@\gamma$-Al_2O_3 **(b)** substrates. Surface AFM images of ZrO_2 **(c)** and $ZrO_2@\gamma$-Al_2O_3 **(d)** substrates. Corresponding height profiles of ZrO_2 **(e)** and $ZrO_2@\gamma$-Al_2O_3 **(f)** substrates.

The water contact angle (WCA) values as shown in Supplementary Fig. 8 are 32 ± 1 and 21 ± 2° for ZrO_2 and ZrO_2@γ-Al_2O_3 substrates, respectively, indicating a more hydrophilic nature after introducing the γ-Al_2O_3 nanoporous interlayer.

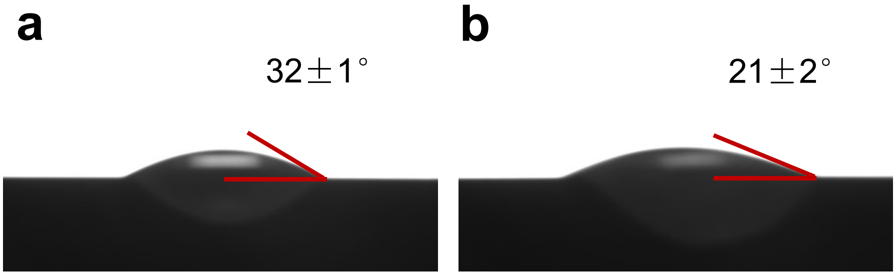

Supplementary Fig. 8 WCA of ZrO_2 **(a)** and ZrO_2@γ-Al_2O_3 **(b)** substrates.

The flexural strength of membrane substrate is 24.96±1.81 MPa is almost equal to that of the tested membranes, since the strength of ultrathin UiO-66 layer can be considered as negligible.

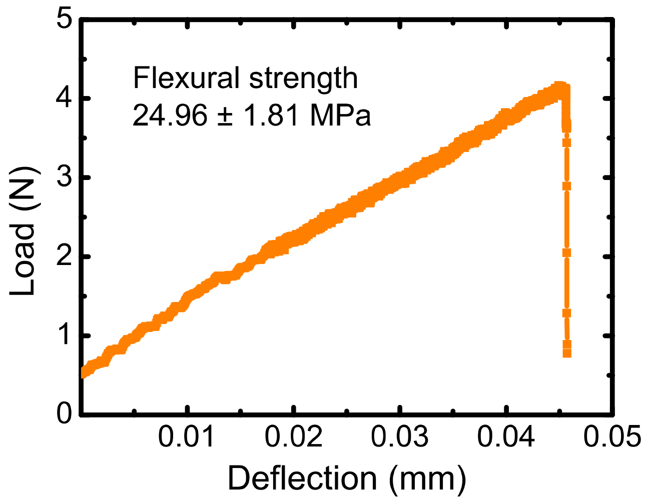

Supplementary Fig. 9 The load-deflection relationship of membrane substrate.

S2.3 Optimization and Performance of ML-UiO-66 Membranes

Supplementary Fig. 10 shows low-quality ML-UiO-66 membranes with poor inter-grown morphology and large inter-crystalline defects were obtained on coarse ZrO_2 substrates when introducing various amounts of CH_3COOH.

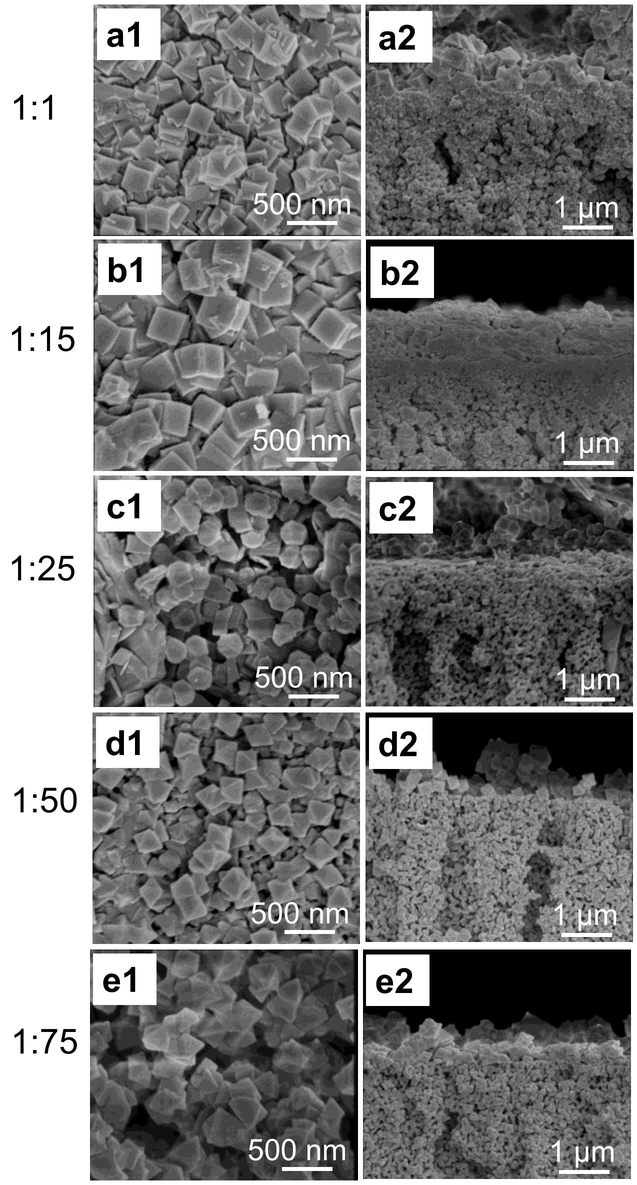

Supplementary Fig. 10 FE-SEM surface (a1, b1, c1, d1, e1) and cross-sectional images (a2, b2, c2, d2, e2) of the ML-UiO-66 membranes synthesized on ZrO_2 substrates under the same operation conditions (Zr^{4+}:CH_3COOH:BDC:DMF = 1:X:1:500, 120°C, 48 h) with different Zr^{4+}/CH_3COOH molar ratios: a1, a2) 1:1, b1, b2) 1:15, c1, c2) 1:25, d1, d2) 1:50, e1, e2) 1:75.

In contrast, when using the ZrO_2@γ-Al_2O_3 substrate, the quality of the achieved membrane improved significantly due to the high-density nucleation site with abundant surface –OH groups (Supplementary Fig. 6). CH_3COOH can more readily complex with Zr clusters. During the ML-UiO-66 membrane growth, CH_3COOH could coordinate with Zr clusters, which plays a competitive role over dicarboxylic acid (BDC)[24], leading to weakly coordination bonding between BDC and Zr clusters. The amount of nucleation decreases with the increase of CH_3COOH, and the morphology of the membrane layer changed from tetrahedron to octahedron (Supplementary Figs. 10 and 11), which is attributed to the coordination modulator mechanism[25]. In our study, when the molar ratio of Zr^{4+}/CH_3COOH was controlled at ~25, an ultrathin and dense ML-UiO-66 membrane was obtained as the optimum morphology and performance (Supplementary Fig. 11).

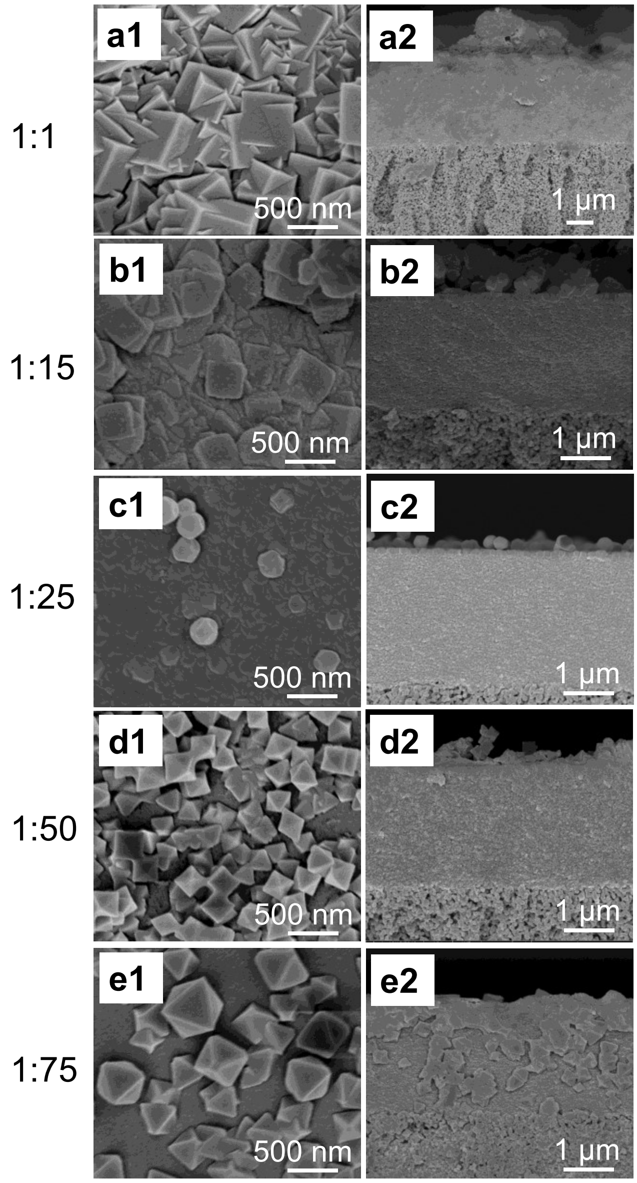

Supplementary Fig. 11 FE-SEM surfaces (a1, b1, c1, d1, e1) and cross-sectional images (a2, b2, c2, d2, e2) of the ML-UiO-66 membranes synthesized on ZrO_2@γ-Al_2O_3 substrates under the same operation conditions (Zr^{4+}:CH_3COOH:BDC:DMF = 1:X:1:500, 120°C, 48 h) with different Zr^{4+}/CH_3COOH molar ratios: a1, a2) 1:1, b1, b2) 1:15, c1, c2) 1:25, d1, d2) 1:50, e1, e2) 1:75.

Supplementary Fig. 12 implies that the ML-UiO-66 membrane grown on the ZrO_2@γ-Al_2O_3 substrate has lower roughness (R_a = ~47 ± 27 nm) than that (R_a = ~133 ± 2 nm) on the ZrO_2 substrate.

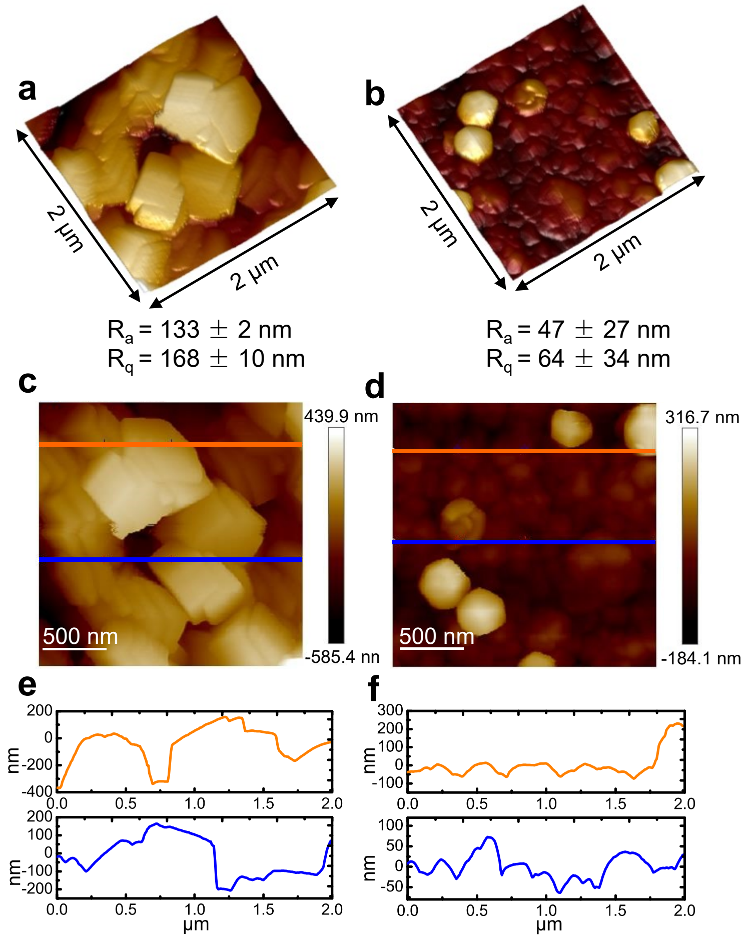

Supplementary Fig. 12 3D AFM images (with R_a (average roughness) and R_q (root-mean-square roughness)) of the ML-UiO-66 membranes grown on ZrO_2 **(a)** and ZrO_2@γ-Al_2O_3 **(b)** substrates. Surface AFM images **(c, d)** and corresponding height profiles (**e, f**) of the ML-UiO-66 membranes grown on ZrO_2 **(c, e)** and ZrO_2@γ-Al_2O_3 **(d, f)** substrates.

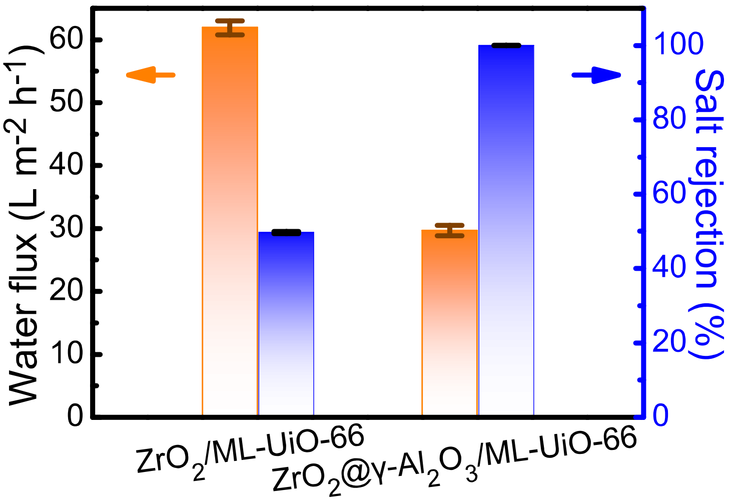

Supplementary Fig. 13 Water flux and salt rejection of ML-UiO-66 membranes grown on ZrO_2 and ZrO_2@γ-Al_2O_3 substrates during PV operation. (Numbers that follows the ±signs are standard deviation (SD) in this study.)

Supplementary Table 3 Comparison of fabrication method and membrane thickness of UiO-66 series membranes.

**Membrane**	**Substrate**	**Fabrication method**	**Membrane thickness （μm）**	**Refs.**
UiO-66-$(OH)_2$	Hollow fiber α-Al_2O_3	Second growth and PSDH*	3.5	[26]
UiO-66	Tublar α-Al_2O_3	Solvothermal method	0.5 – 1.0	[27]
UiO-66	Hollow fiber α-Al_2O_3	Solvothermal method	2.0	[28]
UiO-66	Hollow fiber YSZ	Solvothermal method	1.0	[29]
UiO-66	Disc AAO	Third growth and PSDH	0.8	[30]
UiO-66-NH_2	Tublar α-Al_2O_3	Second growth	1.0	[31]
UiO-66	Hollow fiber ZrO_2@γ-Al_2O_3	Solvothermal method	0.12	This work
ML-UiO-66	Hollow fiber ZrO_2@γ-Al_2O_3	Solvothermal method	0.10	This work

*PSDH: Postsynthetic defect healing

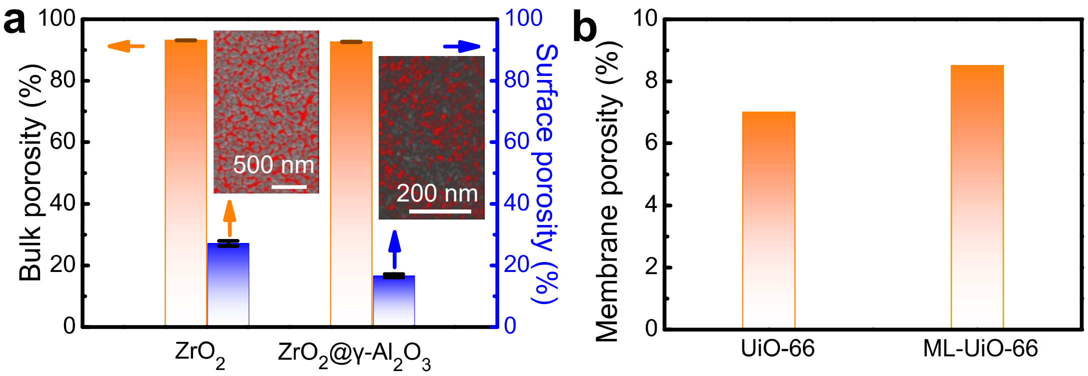

Supplementary Fig. 14 The bulk porosity and surface porosity of ZrO_2 substrate and γ-Al_2O_3 interlayer **(a)** and simulated porosities for UiO-66 and ML-UiO-66 membranes using zeo++ software **(b)**. (Numbers that follows the ±signs are standard deviation (SD) in this study.)

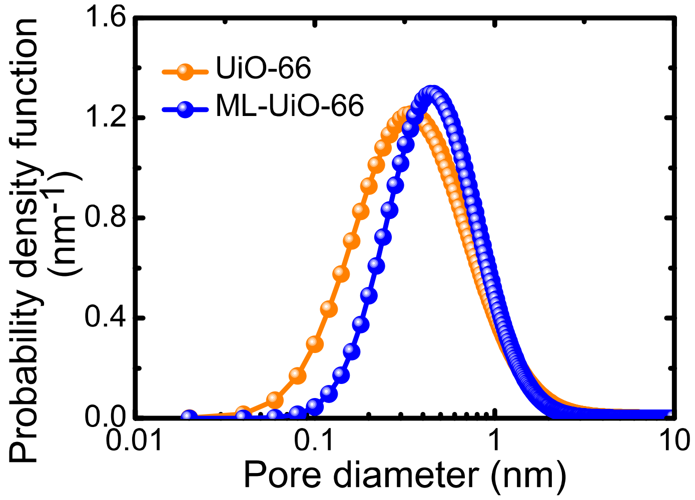

Supplementary Fig. 15 Pore size distribution of UiO-66 and ML-UiO-66 membranes based on the solute rejection method.

The ideal selectivities of H_2/CH_4 for UiO-66 and ML-UiO-66 membranes are experimentally determined to be 30.5 and 17.1, both of which are much higher than the Knudsen diffusion coefficient (2.8). This indicates the membrane integrity had negligible inter-crystalline defects for UiO-66 and ML-UiO-66 membranes.

Supplementary Table 4 The single-gas permeation results of UiO-66 and ML-UiO-66 membranes.

**Membrane**	**H_2 permeance (GPU)**	**CH_4 permeance (GPU)**	**$\alpha_{H2/CH4}$ (ideal)**	**$\alpha_{H2/CH4}$ (Knudson)**
UiO-66	1864.1	61.1	30.5	2.8
ML-UiO-66	662.7	38.7	17.1	2.8

For RO desalination, as shown in Supplementary Table 5, the rejection of NaCl for UiO-66 and ML-UiO-66 membranes were 48.6% and 46.5%, respectively. The aperture sizes of UiO-66 and ML-UiO-66 membranes were experimentally determined to be 5.86 and 6.28 Å (Supplementary Fig. 15), both of which are between H_2O (2.8 Å) and hydrated Na^+ (7.2 Å). Theoretically, both UiO-66 and ML-UiO-66 membranes are able to reject hydrated Na^+. However, low rejection of NaCl can be also similarly observed for UiO-66 (47.0%)[28] and UiO-66-(OH) membranes (45.0%)[26] in previous reports. Such abnormally low salt rejection can be ascribed to the dehydration or partial dehydration effect during the RO desalination process, which has been well confirmed in polymeric[32] and MOF nanochannels[33]. The hydrated and fully dehydrated ionic diameter of Na^+ are 7.16 and 1.90 Å, respectively[33]. Such a dehydration or partial dehydration effect occurred, where Na^+ ions should undergo a dehydration or partial dehydration process to enter the nanochannels of UiO-66 or ML-UiO-66 membranes under a high pressure driven process, and then rehydrated by water molecules when they exit the membrane pore into aqueous media[32,33].

Supplementary Table 5 Desalination performance of UiO-66 and ML-UiO-66 membranes (0.20 wt.% NaCl was applied as feeds at 20 ± 2°C under a pressure difference of 3.0 bar.).

**Membrane**	**Flux (L m^{-2} h^{-1} bar^{-1})**	**Rejection (%)**
UiO-66	0.308	48.6%
ML-UiO-66	0.324	46.5%

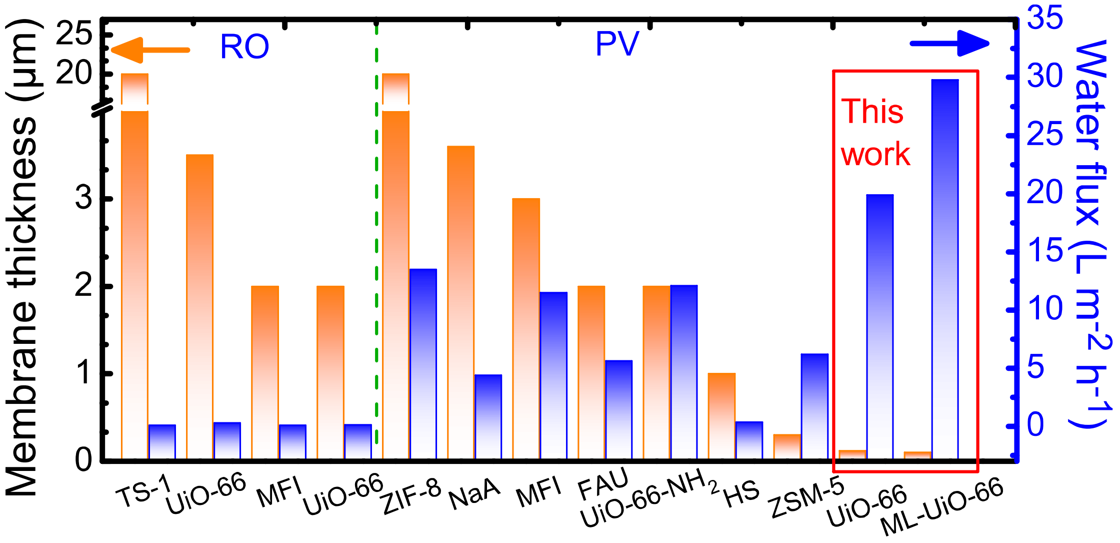

Supplementary Fig. 16 Comparison of membrane thickness and pure water flux of ultrathin UiO-66 and ML-UiO-66 membranes (our study) with other reported thickness zeolite and MOF membranes.

Supplementary Fig. 17 shows that high-quality ultrathin compact membrane morphology with a thickness of only 120 ± 20 nm could also be obtained on the ZrO_2@γ-Al_2O_3 substrate even without the addition of CH_3COOH modulator.

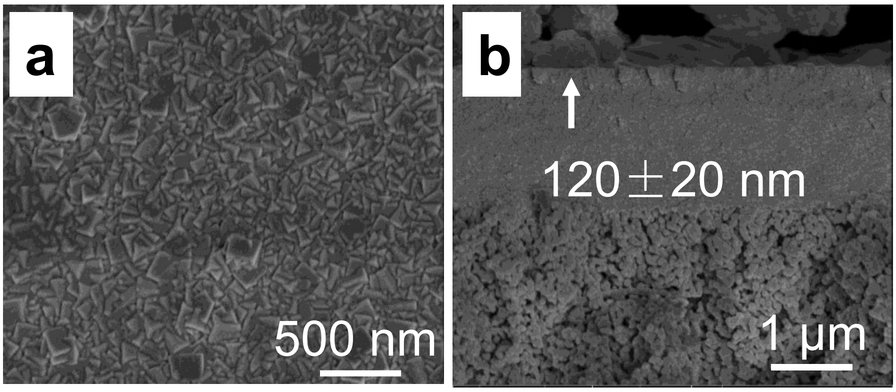

Supplementary Fig. 17 (a) Surface FE-SEM image and **(b)** cross-sectional FE-SEM image of the UiO-66 membrane grown on ZrO_2@γ-Al_2O_3 substrate without the addition of CH_3COOH modulator.

Supplementary Table 6 Comparison in PV desalination performance between UiO-66 and ML-UiO-66 membranes (with CH_3COOH modulator) (feed: 35 g L^{-1} NaCl aqueous solution).

**Membrane type**	**Thickness (μm)**	**Feed temperature (°C)**	**Water flux (L m^{-2} h^{-1})**	**Permeate conductivity (μs cm^{-1})**	**Rejection (%)**
UiO-66	0.12	70	19.9	5.10	99.99
ML-UiO-66	0.10	70	29.8	8.56	99.99

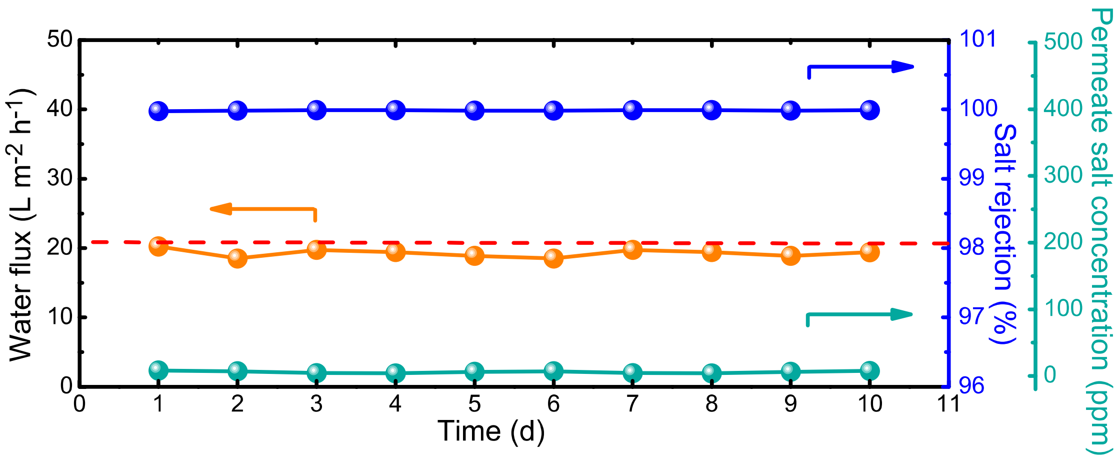

Supplementary Fig. 18 Long-term operating desalination performance (water flux, salt rejection and permeate salt concentration) of UiO-66 membrane for the treatment of 35 g L^{-1} NaCl solution for 240 h (10 days) at 70°C without cleaning. (The red dashed line represents the limit ($Na^{+} < 200$ ppm) of drinking-water quality (fourth edition) issued by the World Health Organization (WHO))

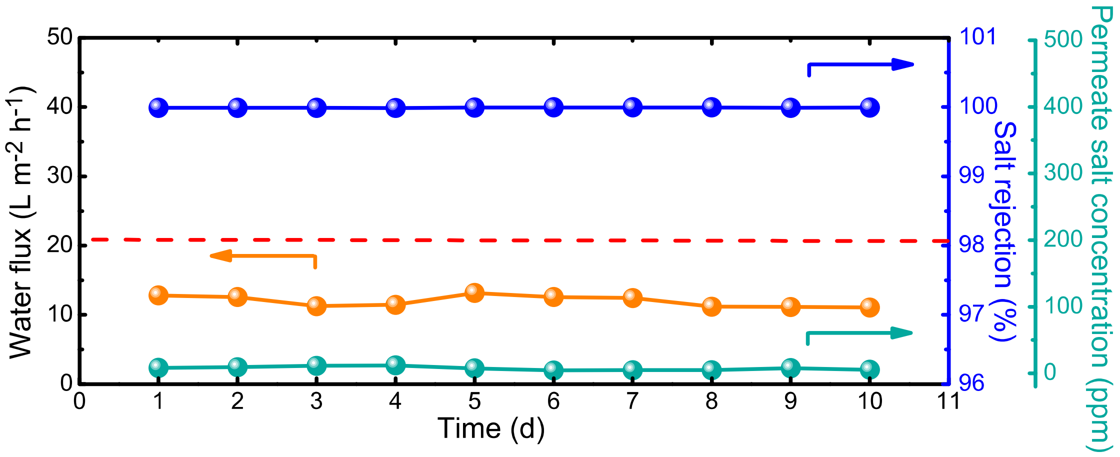

Supplementary Fig. 19 Long-term operating desalination performance (water flux, salt rejection and permeate salt concentration) of UiO-66 membrane for the treatment of hypersaline water (80 g L^{-1}) NaCl solution for 240 h (10 days) at 70°C without cleaning. (The red dashed line represents the limit ($Na^{+} < 200$ ppm) of drinking-water quality (fourth edition) issued by the World Health Organization (WHO))

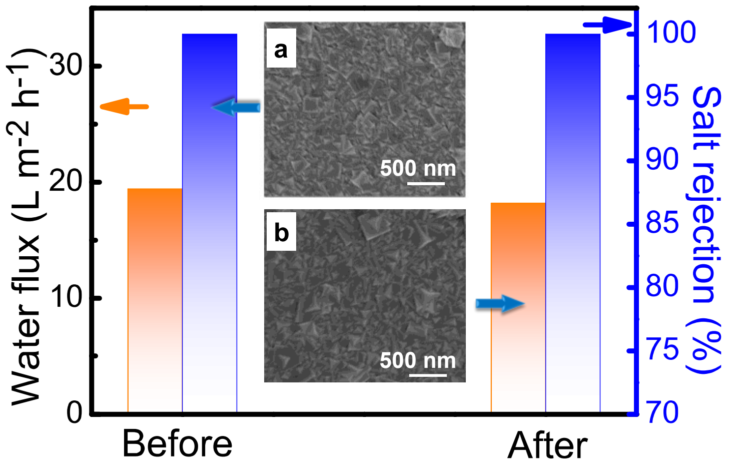

Supplementary Fig. 20 Water flux and salt rejection of ultrathin UiO-66 membranes before and after immersion into NaClO solution (1000 ppm) for 240 h (10 days) for an accelerated chlorine-resistance test. The inset shows FE-SEM images of UiO-66 membrane surface before (a) and after (b) the chlorine-resistance test. (PV conditions: feed 35 g L^{-1} NaCl solution, temperature: 70°C)

Supplementary Fig. 21 shows that the WCA values of UiO-66 and ML-UiO-66 membranes are 83 ± 2 and 50 ± 5°, respectively, experimentally indicating a more hydrophilic feature after introducing missing-linker defects.

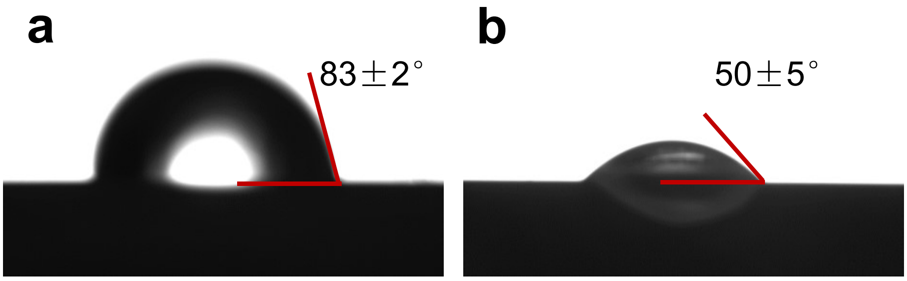

Supplementary Fig. 21 WCA of UiO-66 **(a)** and ML-UiO-66 **(b)** membranes.

At a relative humidity of 95% (25°C), the water adsorption capacities of UiO-66 and ML-UiO-66 are 19.82 and 33.1 wt.%, respectively (Supplementary Fig. 22). Such an enhancement of 67.2% indicates that the introduction of missing-linker defects in the ML-UiO-66 structure enhances its water molecule adsorption ability. Also, at different temperatures, the water adsorption behaviors of UiO-66 and ML-UiO-66 have a similar variation tendency. The initial isosteric heat of water adsorption of ML-UiO-66 is higher than UiO-66 (51.3 vs. 31.9 kJ mol^{-1}, Supplementary Fig. 22d), which implies a more favorable interaction between ML-UiO-66 and water molecules than UiO-66. That is to say, ML-UiO-66 has better water adsorption ability than UiO-66[19].

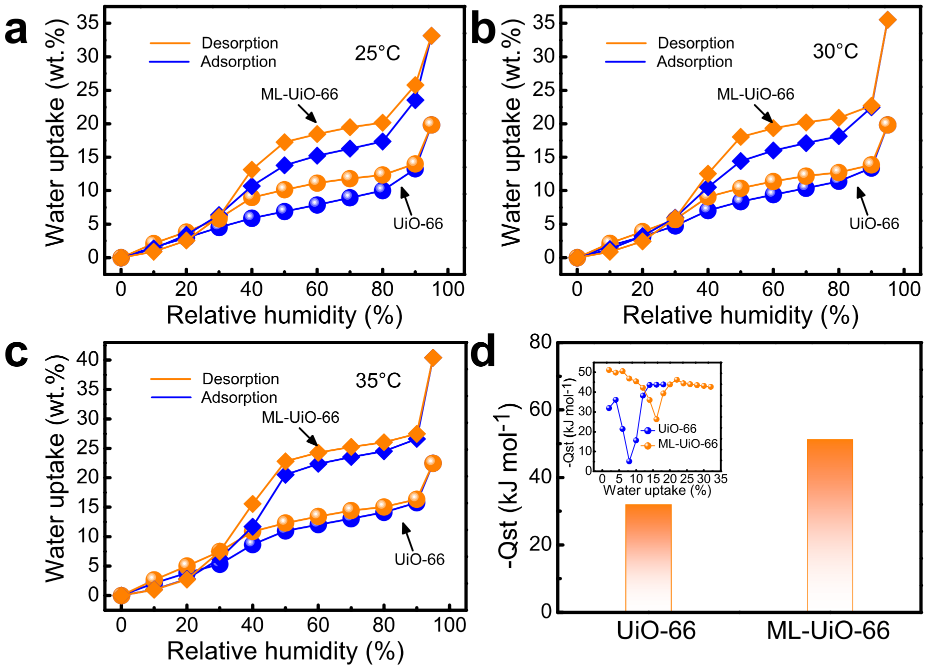

Supplementary Fig. 22 Water uptake behavior of UiO-66 and ML-UiO-66 structures. Water adsorption isotherms of UiO-66 and ML-UiO-66 measured at 25°C **(a)**, 30°C **(b)** and 35°C **(c)**. **(d)** Initial isosteric heat of water adsorption (Q_{st}) of UiO-66 and ML-UiO-66 (the inset shows the Q_{st} of UiO-66 and ML-UiO-66 as a function of water uptake capacity).

Supplementary Table 7 Adsorption, water diffusivity and permeability of ceramic-based UiO-66 and ML-UiO-66 membranes.

**Membrane**	**Adsorption (-)**	**Diffusivity ($\times 10^{-6}$ cm^2 s^{-1})**	**Permeability ($\times 10^{-6}$ cm^2 s^{-1}) (P=D$\times$S)**
UiO-66	0.40	40.22	160.86
ML-UiO-66	0.43	48.49	208.52

Supplementary Table 8 Comparison of activation energy for different thermal-driven membrane processes such as MD and PV between the ML-UiO-66 membrane fabricated in this work and those reported in the literatures.

**Membrane process**	**Feed concentration ($NaCl$, g L^{-1})**	**Membrane**	**Activated energy (KJ mol^{-1})**	**Refs.**
DCMD*	35	Spinel-CNTs#	63.2	[34]
MD**	40	YSZ-FAS##	38.2	[35]
DCMD	5	Zirconia	46.1	[36]
AGMD***	5	Titania	30.4	[36]
VMD	40	Al_2O_3-FAS	45.8	[37]
PV	35	UiO-66-NH_2	46.8	[31]
PV	35	ZIF-8	10.5	[38]
PV	35	NaA	37.4	[39]
PV	35	FAU###	29.5	[40]
PV	35	ML-UiO-66	24.8	This work

*: Direct contact membrane distillation; **: Membrane distillation; ***: Air gap membrane distillation; #: Carbon nanotubes; ##: Fluoralkylsilanes; ###: Faujasite.

Supplementary Fig. 23 shows that the membrane surface morphology (a) and phase (b) of the ML-UiO-66 membrane were not altered after 10-day desalination operation.

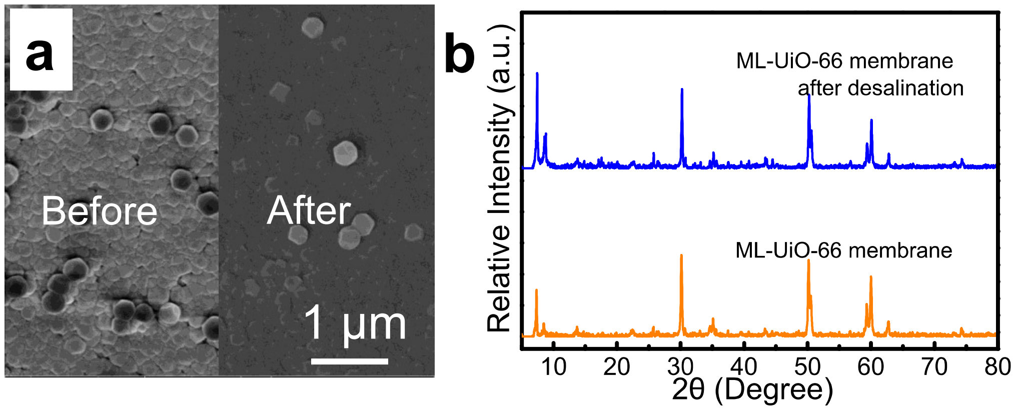

Supplementary Fig. 23 **(a)** Surface FE-SEM images and **(b)** XRD patterns of ML-UiO-66 membrane before and after 10-day desalination operation (feed concentration: 35 g L^{-1}, feed temperature: 70°C).

Supplementary Table 9 Performance comparison between the ultrathin ML-UiO-66 membrane (our work) and existing state-of-the-art inorganic desalination membranes such as zeolite and MOFs.

Membrane type	Membrane process	Operating pressure (bar)	Operating temperature (°C)	Salt concentration (wt.%)	Water flux (L m^{-2} h^{-1})	Rejection (%)	Operation (h)	Refs.
MFI	RO	20	20	0.6	0.1	76	150	41
MFI	RO	7	20	0.3	0.1	95	4320	42
TS-1	RO	14	20	0.6	0.1	5	80	43
ZSM-5	RO	20	20	0.6	1.129	92.9	--	44
UiO-66	RO	3	20	0.2	0.286	45	170	26
UiO-66	RO	10	20	0.2	0.14	47	--	28
PVDF	MD	--	70	3.5	61.9	99.9	--	45
PTFE	MD	--	75	3.5	6.5	99.9	--	Commercial*
CNTs	MD	--	60	3.5	37	99.9	18	34
CNTs	MD	--	75	3.5	43.2	99.9	12	3
Organosilica	MD	--	60	3.5	13	99.9	2.33	46
MOF-Al_2O_3	MD	--	90	3.5	32.3	--	--	47
GO	PV	~1	70	3.5	124	99	168	48
MFI	PV	~1	80	3.8	0.7	97	26	49
MFI	PV	~1	75	0.3	11.5	99	100	50
HS	PV	~1	90	1	0.35	>99.5	100	51
ZSM-5	PV	~1	80	3	6.2	>99.5	100	52
FAU	PV	~1	90	3.5	5.64	99	120	40

NaA	PV	~1	69	0.6	1.90	99.9	4	[39]
NaA	PV	~1	77	0.6	4.4	99.9	4	[39]
NaA	PV	~1	20	0.6	1.43	99.83	4	[39]
UiO-66-NH_2	PV	~1	90	3.5	12.1	99.7	120	[31]
ZIF-8	PV	~1	25	2.0	5.8	99.8	168	[38]
ZIF-8	PV	~1	50	2.0	8.1	99.8	168	[38]
ZIF-8	PV	~1	25	2.0	10.8	99.8	168	[38]
ZIF-8	PV	~1	25	2.0	13.5	99.8	168	[38]
ML-UiO-66	PV	~1	70	3.5	29.8	99.5	240	This work

* Nanjing Cas-Bidun Newmem Technology Co., Ltd.

S2.4 Characterization of Missing-linker Defect

The N_2 adsorption-desorption isotherms of UiO-66 and ML-UiO-66 powder are shown in Supplementary Fig. 24, where the calculated specific surface area, pore volume and pore size are summarized in Supplementary Table 10. Both the UiO-66 and ML-UiO-66 samples had similar type-I behaviors, which indicate their microporous characteristic similar with the results reported in the literature[28].

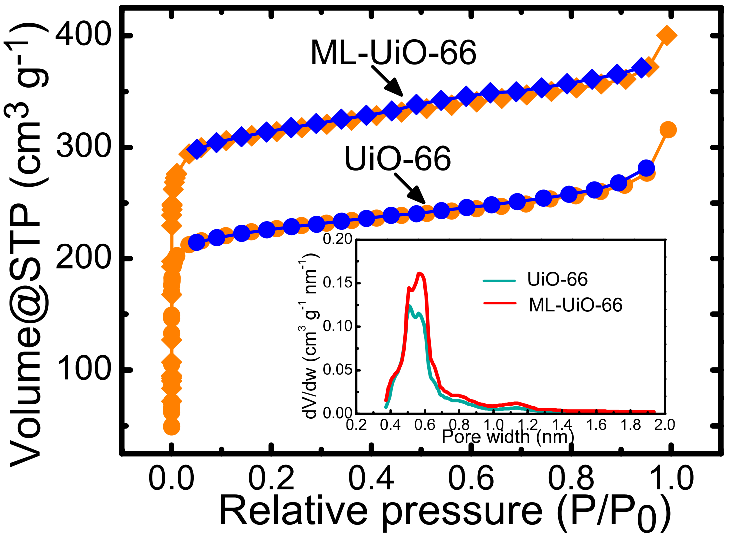

Supplementary Fig. 24 Comparison of the experimentally-determined nitrogen adsorption-desorption isotherms of UiO-66 and ML-UiO-66 powders (the inset shows the pore width distribution of UiO-66 and ML-UiO-66).

Supplementary Table 10 Specific surface area, pore volume and pore size of UiO-66 and ML-UiO-66 calculated from experimental nitrogen adsorption-desorption isotherms (Supplementary Fig. 24).

Material	Specific surface area (m^2 g^{-1})	Pore volume (cm^3 g^{-1})	Pore size (nm)
UiO-66	990.4	0.488	0.508
ML-UiO-66	1249.0	0.619	0.568

TG data can be applied to quantitatively analyze the density of missing-linker defects[8,53]. Ideally, the dihydroxylation reaction of UiO-66 (i.e., $Zr_6O_6(BDC)_6$ follows Supplementary Eq. 10.

$$Zr_6O_6(BDC)_6\ (s) + 45O_2\ (g) \rightarrow 6ZrO_2\ (s) + 48CO_2\ (g) + 12H_2O\ (g) \tag{10}$$

Since the molar masses of $Zr_6O_6(BDC)_6$ and ZrO_2 are 1628.03 g mol^{-1} and 123.22 g mol^{-1}, respectively, the mass ratio of $Zr_6O_6(BDC)_6$: ZrO_2 is calculated to be 2.202. If final ZrO_2 mass is normalized to 100%, the TG plateau value ($T_{plat.}$) of the ideal defect-free UiO-66 structure should be 220.2%.

Assuming x is the number of missing-linker per Zr_6 formula unit, a missing-linker defective composition can be expressed as $Zr_6O_{6+x}(BDC)_{6-x}$. For UiO-66 (Supplementary Fig. 25a), the specific calculation process is as Supplementary Eq. 11 and Supplementary Eq. 12.

$$Wt.PL_{Theo.} = \frac{W_{Ideal.Plat.} - W_{End}}{NL_{Ideal}} = \frac{(220.2 - 100)}{6} \times 100\% = 20.03\% \tag{11}$$

$$NL_{Exp.} = 6 - x = \frac{W_{Exp.Plat.} - W_{End}}{Wt.PL_{Theo.}} = \frac{(220.2 - 100)\%}{20.03\%} = 5.99 \tag{12}$$

$x = 0.01$

where, $Wt.PL_{Theo.}$ is the weight contribution per BDC linker, $W_{Ideal.Plat.}$ and $W_{Exp.Plat.}$ are respectively the TGA plateau of the ideal dehydroxylated and the experimental UiO-66 materials. W_{end} is the end weight of the TGA curve (100%). $NL_{Exp.}$ is linker number of the experimental UiO-66 data. Here NL_{Ideal} is 6.

For quantitative analysis of ML-UiO-66 (Supplementary Fig. 25b), $W_{Exp.Plat.}$ = 191.9%, the specific calculation process is as Supplementary Eq. 13.

$$NL_{Exp.} = 6 - x = \frac{W_{Exp.Plat.} - W_{End}}{Wt.PL_{Theo.}} = \frac{(191.9 - 100)\%}{20.03\%} = 4.59 \tag{13}$$

$x = 1.41$

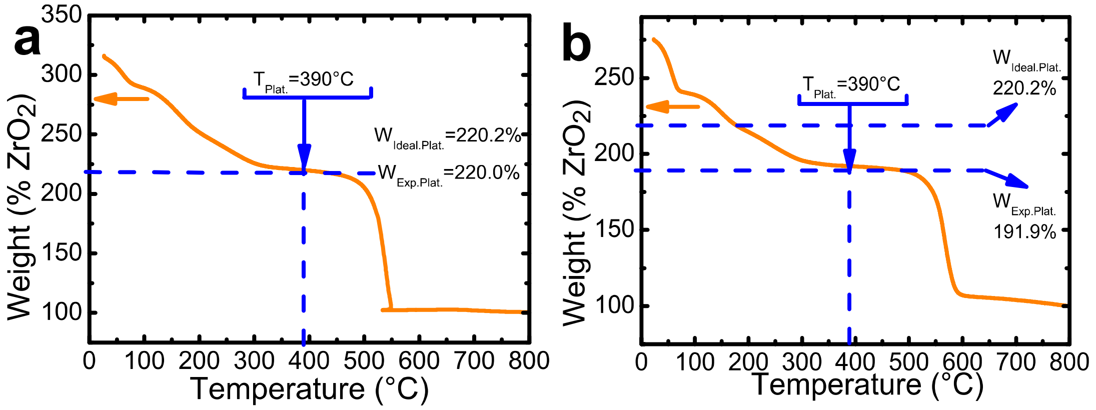

Supplementary Fig. 25 TG curves of UiO-66 **(a)** and ML-UiO-66 **(b)** between 25 and 800°C at a constant heating rate of 10°C min^{-1}.

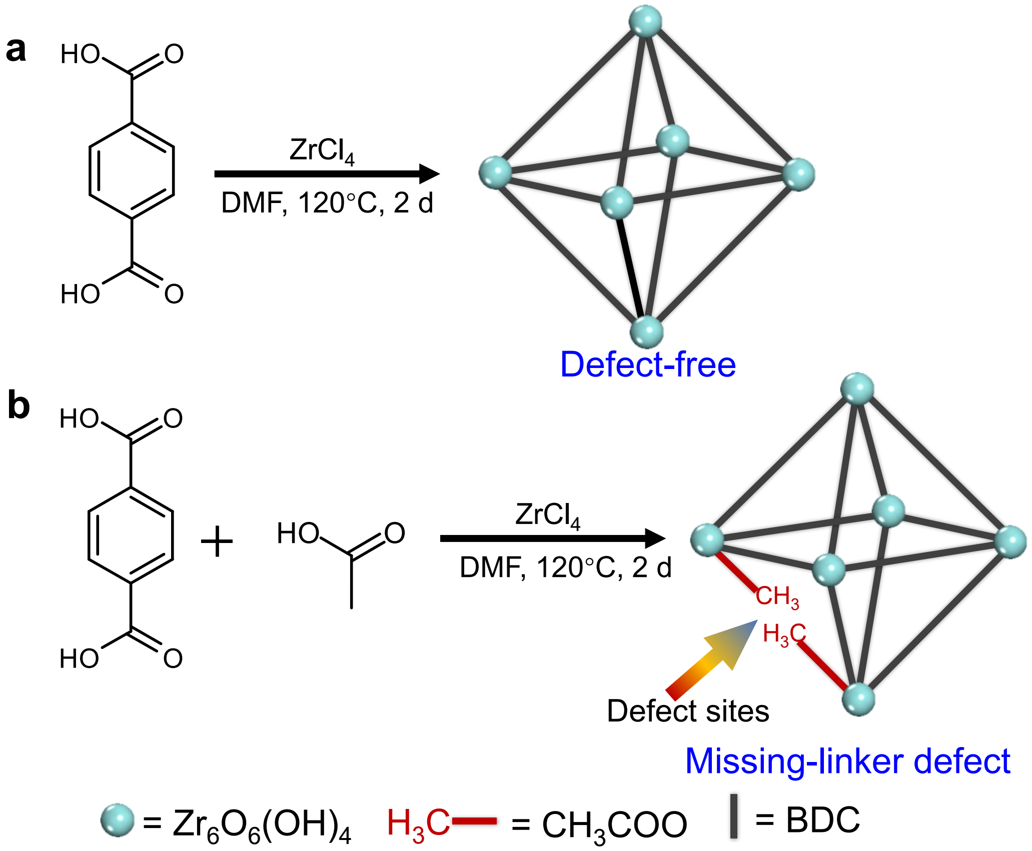

Supplementary Fig. 26 The routes toward the synthesis of UiO-66 **(a)** and ML-UiO-66 **(b)**.

S2.5 Computational Modeling

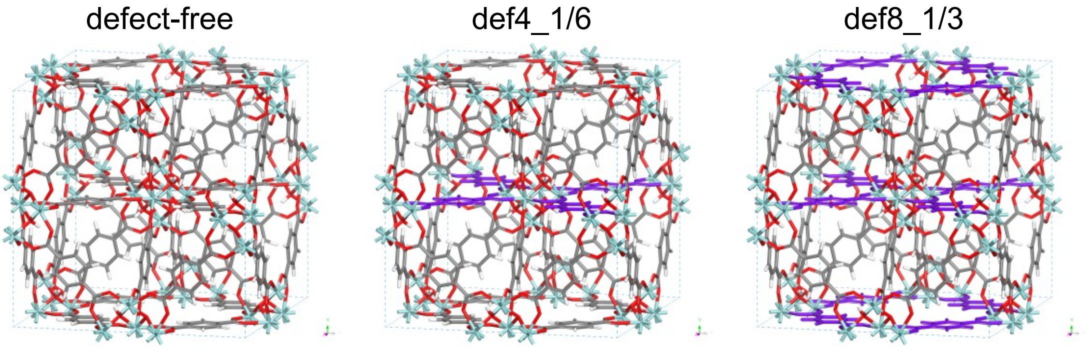

Supplementary Fig. 27 Structural illustrations of the defect-free and two defective UiO-66 unit cells with different defect densities considered in this work. UiO-66 structures are represented as sticks with color codes of carbon: grey, oxygen: red, hydrogen: white, and zirconium: cyan. Missing linkers are highlighted in purple. The first number in the structure name indicates how many linkers were removed per unit cell (e.g., def4: a UiO-66 structure with four missing linkers per unit cell). Additionally, the number after the underscore refers to the corresponding defect density of each defective structure.

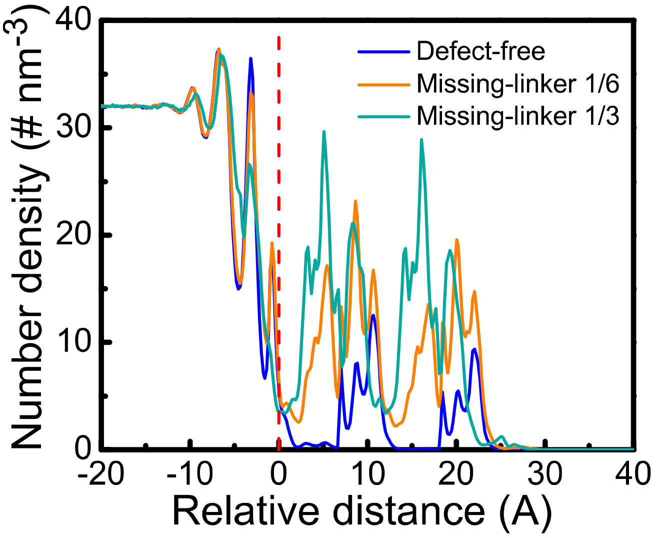

Supplementary Fig. 28 Water density profiles of defect-free and defective UiO-66 membranes along the permeation direction.

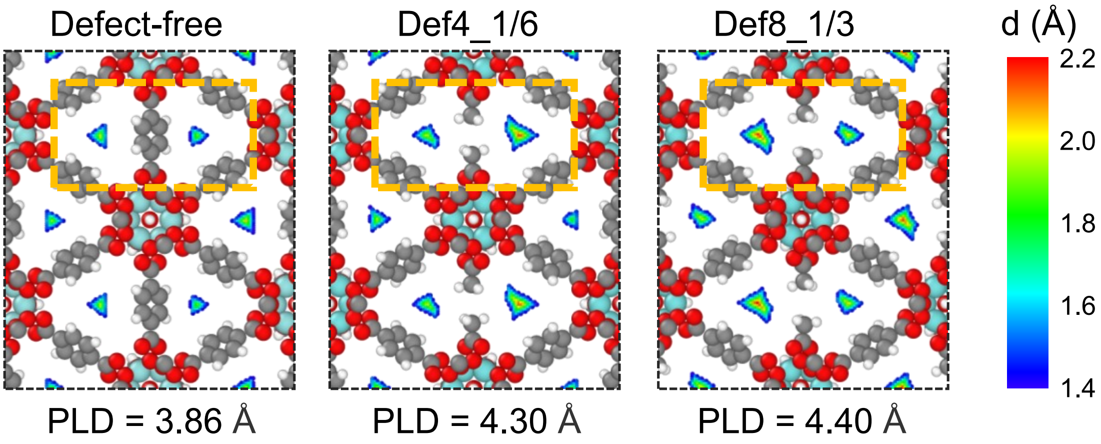

Supplementary Fig. 29 Two-dimensional accessible area maps of the triangle pore windows for the defect-free structure and two defective UiO-66 structures with different defect densities (i.e., missing-linker_1/6 and missing-linker_1/3). Color code represents the distance between the accessible grid and its nearest framework atom. Warmer color indicates a larger distance. PLD refers to as the pore limiting diameter, indicating the diameter of the largest sphere that can transverse the membrane. UiO-66 structures are represented as balls with color codes of carbon: grey, oxygen: red, hydrogen: white, and zirconium: cyan.

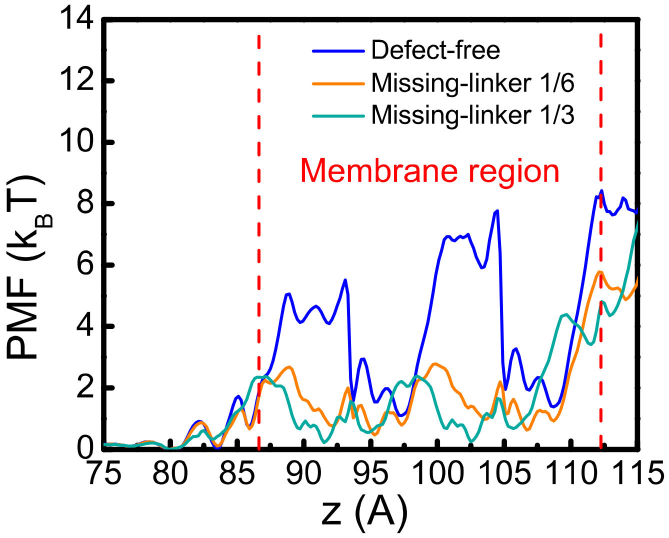

Supplementary Fig. 30 Potential of mean force (PMF) profiles of water molecules along the permeation direction.

Supplementary References

1. Zhu, L., Chen, M., Dong, Y., Tang, C. Y., Huang, A., Li, L. A low-cost mullite-titania composite ceramic hollow fiber microfiltration membrane for highly efficient separation of oil-in-water emulsion. *Water Res.* **90,** 277-285 (2016).
2. Zhang, M., Jin, W., Yang, F., Duke, M., Dong, Y., Tang, C. Y. Engineering a nanocomposite interlayer for a novel ceramic-based forward osmosis membrane with enhanced performance. *Environ. Sci. Technol.* **54,** 7715-7724 (2020).
3. Si, Y., Sun, C., Li, D., Yang, F., Tang, C. Y., Quan, X., Dong, Y., Guiver, M. D. Flexible superhydrophobic metal-based carbon nanotube membrane for electrochemically enhanced water treatment. *Environ. Sci. Technol.* **54,** 9074-9082 (2020).
4. Wang, X., Li, Y., Yu, H., Yang, F., Tang, C. Y., Quan, X., Dong, Y. High-flux robust ceramic membranes functionally decorated with nano-catalyst for emerging micro-pollutant removal from water. *J. Membr. Sci.* **611,** 118281 (2020).
5. Zou, C., Lin, L. C. Exploring the potential and design of zeolite nanosheets as pervaporation membranes for ethanol extraction. *Chem. Commun.* **54,** 13200-13203 (2018).
6. Zou, C., Lin, L.-C. Potential and design of zeolite nanosheets as pervaporation membranes for ethanol extraction. *Ind. Eng. Chem. Res.* **59,** 12845-12854 (2020).
7. Virmani, E., Rotter, J. M., Mahringer, A., von Zons, T., Godt, A., Bein, T., Wuttke, S., Medina, D. D. On-surface synthesis of highly oriented thin metal–organic framework films through vapor-assisted conversion. *J. Am. Chem. Soc.* **140,** 4812-4819 (2018).
8. Shearer, G. C., Chavan, S., Bordiga, S., Svelle, S., Olsbye, U., Lillerud, K. P. Defect engineering: tuning the porosity and composition of the metal–organic framework UiO-66 via modulated synthesis. *Chem. Mater.* **28,** 3749-3761 (2016).
9. Wu, H., Chua, Y. S., Krungleviciute, V., Tyagi, M., Chen, P., Yildirim, T., Zhou, W. Unusual and highly tunable missing-linker defects in zirconium metal–organic framework UiO-66 and their important effects on gas adsorption. *J. Am. Chem. Soc.* **135,** 10525-10532 (2013).
10. Liu, L., Chen, Z., Wang, J., Zhang, D., Zhu, Y., Ling, S., Huang, K. W., Belmabkhout, Y., Adil, K., Zhang, Y., Slater, B., Eddaoudi, M., Han, Y. Imaging defects and their evolution in a metal–organic framework at sub-unit-cell resolution. *Nat. Chem.* **11,** 622-628 (2019).
11. Mayo, S. L., Olafson, B. D., Goddard, W. A. DREIDING: a generic force field for molecular simulations. *J. Phys. Chem.* **94,** 8897-8909 (1990).
12. A. K. Rappé, C. J. Casewit, K. S. Colwell, W. A. Goddard III, Skiff, W. M. UFF, a full periodic table force field for molecular mechanics and molecular dynamics simulations. *J. Am. Chem. Soc.* **114,** 10024-10035 (1992).
13. Stewart, J. J. Optimization of parameters for semiempirical methods VI: more modifications to the NDDO approximations and re-optimization of parameters. *J. Mol. Model.* **19,** 1-32 (2013).
14. Hoover, W. G. Canonical dynamics: equilibrium phase-space distributions. *Phys. Rev. A* **31,** 1695-1697 (1985).
15. Berendsen, H. J. C., Grigera, J. R., Straatsma, T. P. The missing term in effective pair potentials. *J. Phys. Chem.* **91,** 6269-6271 (1987).
16. In Suk Joung, Thomas E. Cheatham, I. Determination of alkali and halide monovalent ion

parameters for use in explicitly solvated biomolecular simulations. *J. Phys. Chem. B* **112,** 9020-9041 (2008).

17. Ma, D., Han, G., Gao, Z. F., Chen, S. B. Continuous UiO-66-type metal–organic framework thin film on polymeric support for organic solvent nanofiltration. *ACS Appl. Mater. Interfaces* **11,** 45290-45300 (2019).
18. Li, B., Japip, S., Lai, J.-Y., Chung, T.-S. Revitalize integrally skinned hollow fiber membranes with spatially impregnated 3D-macrocycles for organic solvent nanofiltration. *Chem. Eng. J.* **422,** 130015 (2021).
19. Lee, T. H., Jung, J. G., Kim, Y. J., Roh, J. S., Yoon, H. W., Ghanem, B. S., Kim, H. W., Cho, Y. H., Pinnau, I., Park, H. B. Defect engineering in metal–organic framework towards advanced mixed matrix membranes for efficient propylene-propane separation. *Angew. Chem. Int. Ed.* **60,** 13081-13088 (2021).
20. Lee, T. H., Oh, J. Y., Jang, J. K., Moghadam, F., Roh, J. S., Yoo, S. Y., Kim, Y. J., Choi, T. H., Lin, H., Kim, H. W., Park, H. B. Elucidating the role of embedded metal–organic frameworks in water and ion transport properties in polymer nanocomposite membranes. *Chem. Mater.* **32,** 10165-10175 (2020).
21. Manjumol, K. A., Shajesh, P., Baiju, K. V., Warrier, K. G. K. An 'Eco-friendly' all aqueous sol gel process for multi functional ultrafiltration membrane on porous tubular alumina substrate. *J. Membr. Sci.* **375,** 134-140 (2011).
22. Setiawan, H., Khairani, R., Rahman, M. A., Septawendar, R., Mukti, R. R., Dipojono, H. K., Purwasasmita, B. S. Synthesis of zeolite and γ-alumina nanoparticles as ceramic membranes for desalination applications. *J. Aust. Ceram. Soc.* **53,** 531-538 (2017).
23. Gu, Y., Meng, G. A model for ceramic membrane formation by dip-coating. *J. Eur. Ceram. Soc.* **19,** 1961-1966 (1999).
24. Piszczek, P., Radtke, A., Grodzicki, A., Wojtczak, A., Chojnacki, J. The new type of $[Zr_6(\mu_3\text{-}O)_4(\mu_3\text{-}OH)_4]$ cluster core: crystal structure and spectral characterization of $[Zr_6O_4(OH)_4(OOCR)_{12}]$ (R=Bu^t, $C(CH_3)_2Et$). *Polyhedron* **26,** 679-685 (2007).
25. Schaate, A., Roy, P., Godt, A., Lippke, J., Waltz, F., Wiebcke, M., Behrens, P. Modulated synthesis of Zr-based metal–organic frameworks: from nano to single crystals. *Chemistry* **17,** 6643-6651 (2011).
26. Wang, X., Zhai, L., Wang, Y., Li, R., Gu, X., Yuan, Y. D., Qian, Y., Hu, Z., Zhao, D. Improving water-treatment performance of zirconium metal–organic framework membranes by postsynthetic defect healing. *ACS Appl. Mater. Interfaces* **9,** 37848-37855 (2017).
27. Miyamoto, M., Hori, K., Goshima, T., Takaya, N., Oumi, Y., Uemiya, S. An organoselective zirconium-based metal-organic-framework UiO-66 membrane for pervaporation. *Eur. J. Inorg. Chem.* **14,** 2094-2099 (2017).
28. Liu, X., Demir, N. K., Wu, Z., Li, K. Highly water-stable zirconium metal–organic framework UiO-66 membranes supported on alumina hollow fibers for desalination. *J. Am. Chem. Soc.* **137,** 6999-7002 (2015).
29. Liu, X., Wang, C., Wang, B., Li, K. Novel organic-dehydration membranes prepared from zirconium metal–organic frameworks. *Adv. Funct. Mater.* **27,** 1604311 (2017).
30. Wu, W., Li, Z., Chen, Y., Li, W. Polydopamine-modified metal–organic framework membrane with

enhanced selectivity for carbon capture. *Environ. Sci. Technol.* **53,** 3764-3772 (2019).

31. Wan, L., Zhou, C., Xu, K., Feng, B., Huang, A. Synthesis of highly stable UiO-66-NH_2 membranes with high ions rejection for seawater desalination. *Microporous Mesoporous Mater.* **252,** 207-213 (2017).
32. Lu, C., Hu, C., Ritt, C. L., Hua, X., Sun, J., Xia, H., Liu, Y., Li, D. W., Ma, B., Elimelech, M., Qu, J. In situ characterization of dehydration during ion transport in polymeric nanochannels. *J. Am. Chem. Soc.* **143,** 14242-14252 (2021).
33. Zhang, H., Hou, J., Hu, Y., Wang, P., Ou, R., Jiang, L., Liu, J. Z., Freeman, B. D., Hill, A. J., Wang, H. Ultrafast selective transport of alkali metal ions in metal organic frameworks with subnanometer pores. *Sci. Adv.* **4,** eaaq0066 (2018).
34. Dong, Y., Ma, L., Tang, C. Y., Yang, F., Quan, X., Jassby, D., Zaworotko, M. J., Guiver, M. D. Stable superhydrophobic ceramic-based carbon nanotube composite desalination membranes. *Nano Lett.* **58,** 5514-5521 (2018).
35. Liu, T., Lei, L., Gu, J., Wang, Y., Winnubst, L., Chen, C., Ye, C., Chen, F. Enhanced water desalination performance through hierarchically-structured ceramic membranes. *J. Eur. Ceram. Soc.* **37,** 2431-2438 (2017).
36. Cerneaux, S., Strużyńska, I., Kujawski, W. M., Persin, M., Larbot, A. Comparison of various membrane distillation methods for desalination using hydrophobic ceramic membranes. *J. Membr. Sci.* **337,** 55-60 (2009).
37. Fang, H., Gao, J. F., Wang, H. T., Chen, C. S. Hydrophobic porous alumina hollow fiber for water desalination via membrane distillation process. *J. Membr. Sci.* **403-404,** 41-46 (2012).
38. Zhu, Y., Gupta, K. M., Liu, Q., Jiang, J., Caro, J., Huang, A. Synthesis and seawater desalination of molecular sieving zeolitic imidazolate framework membrane. *Desalination* **385,** 75-82 (2016).
39. Cho, C. H., Oh, K. Y., Kim, S. K., Yeo, J. G., Sharma, P. Pervaporative seawater desalination using NaA zeolite membrane: mechanisms of high water flux and high salt rejection. *J. Membr. Sci.* **371,** 226-238 (2011).
40. Zhou, C., Zhou, J., Huang, A. Seeding-free synthesis of zeolite FAU membrane for seawater desalination by pervaporation. *Microporous Mesoporous Mater.* **234,** 377-383 (2016).
41. Li, L., Dong, J., Nenoff, T. M., Lee, R. Desalination by reverse osmosis using MFI zeolite membranes. *J. Membr. Sci.* **243,** 401-404 (2004).
42. Zhu, B., Hong, Z., Milne, N., Doherty, C. M., Zou, L., Lin, Y. S., Hill, A. J., Gu, X., Duke, M. Desalination of seawater ion complexes by MFI-type zeolite membranes: temperature and long term stability. *J. Membr. Sci.* **453,** 126-135 (2014).
43. Liu, X., Xie, B., Zhang, B., Ma, L. Preparation of hierarchical TS-1 zeolite membrane via a dissolution–recrystallization process. *J. Mater. Chem.* **53,** 1851-1861 (2017).
44. Li, L., Liu, N., McPherson, B., Lee, R. Enhanced water permeation of reverse osmosis through MFI-Type zeolite membranes with high aluminum contents. *2007* **46,** 1584-1589.
45. Lu, K.-J., Zuo, J., Chung, T.-S. Novel PVDF membranes comprising n-butylamine functionalized graphene oxide for direct contact membrane distillation. *J. Membr. Sci.* **539,** 34-42 (2017).
46. Chua, Y. T., Lin, C. X., Kleitz, F., Zhao, X. S., Smart, S. Nanoporous organosilica membrane for water desalination. *Chem. Commun. (Camb)* **49,** 4534-6 (2013).
47. Zuo, J., Chung, T.-S. Metal–organic framework-functionalized alumina membranes for vacuum

membrane distillation. *Water* **8,** 586 (2016).

48. Song, Y., Li, R., Pan, F., He, Z., Yang, H., Li, Y., Yang, L., Wang, M., Wang, H., Jiang, Z. Ultrapermeable graphene oxide membranes with tunable interlayer distances via vein-like supramolecular dendrimers. *J. Mater. Chem. A.* **7,** 18642-18652 (2019).
49. Duke, M. C., O'Brien-Abraham, J., Milne, N., Zhu, B., Lin, J. Y. S., Costa, J. C. D. d. Seawater desalination performance of MFI type membranes made by secondary growth. *Sep. Purif. Technol.* **68,** 343-350 (2009).
50. Drobek, M., Yacou, C., Motuzas, J., Julbe, A., Ding, L., Diniz da Costa, J. C. Long term pervaporation desalination of tubular MFI zeolite membranes. *J. Membr. Sci.* **415-416,** 816-823 (2012).
51. Khajavi, S., Jansen, J. C., Kapteijn, F. Production of ultra pure water by desalination of seawater using a hydroxy sodalite membrane. *J. Membr. Sci.* **356,** 52-57 (2010).
52. Cao, Z., Zeng, S., Xu, Z., Arvanitis, A., Yang, S., Gu, X., Dong, J. Ultrathin ZSM-5 zeolite nanosheet laminated membrane for high-flux desalination of concentrated brines. *Sci. Adv.* **4,** eaau8634 (2018).
53. Shearer, G. C., Chavan, S., Ethiraj, J., Vitillo, J. G., Svelle, S., Olsbye, U., Lamberti, C., Bordiga, S., Lillerud, K. P. Tuned to perfection: ironing out the defects in metal–organic framework UiO-66. *Chem. Mater.* **26,** 4068-4071 (2014).
